# Growth in a biofilm promotes conjugation of a *bla*_NDM-1_-bearing plasmid between *Klebsiella pneumoniae* strains

**DOI:** 10.1128/msphere.00170-23

**Published:** 2023-07-07

**Authors:** Sarah J. Element, Robert A. Moran, Emilie Beattie, Rebecca J. Hall, Willem van Schaik, Michelle M.C. Buckner

**Affiliations:** 1 Institute of Microbiology and Infection, College of Medical and Dental Sciences, University of Birmingham, Birmingham, West Midlands, United Kingdom; JMI Laboratories, North Liberty, Iowa, USA

**Keywords:** carbapenemase, carbapenem-resistance plasmid, *Klebsiella pneumoniae*, conjugation, biofilm, gene expression

## Abstract

Antimicrobial resistance (AMR) is a growing problem, especially in Gram-negative Enterobacteriaceae such as *Klebsiella pneumoniae*. Horizontal transfer of conjugative plasmids contributes to AMR gene dissemination. Bacteria such as *K. pneumoniae* commonly exist in biofilms, yet most studies focus on planktonic cultures. Here we studied the transfer of a multi-drug resistance plasmid in planktonic and biofilm populations of *K. pneumoniae*. We determined plasmid transfer from a clinical isolate, CPE16, which carried four plasmids, including the 119-kbp *bla*_NDM-1_-bearing F-type plasmid pCPE16_3, in planktonic and biofilm conditions. We found that transfer frequency of pCPE16_3 in a biofilm was orders-of-magnitude higher than between planktonic cells. In 5/7 sequenced transconjugants (TCs) multiple plasmids had transferred. Plasmid acquisition had no detectable growth impact on TCs. Gene expression of the recipient and a transconjugant was investigated by RNA-sequencing in three lifestyles: planktonic exponential growth, planktonic stationary phase, and biofilm. We found that lifestyle had a substantial impact on chromosomal gene expression, and plasmid carriage affected chromosomal gene expression most in stationary planktonic and biofilm lifestyles. Furthermore, expression of plasmid genes was lifestyle-dependent, with distinct signatures across the three conditions. Our study shows that growth in biofilm greatly increased the risk of conjugative transfer of a carbapenem resistance plasmid in *K. pneumoniae* without fitness costs and minimal transcriptional rearrangements, thus highlighting the importance of biofilms in the spread of AMR in this opportunistic pathogen.

**IMPORTANCE:**

Carbapenem-resistant *K. pneumoniae* is particularly problematic in hospital settings. Carbapenem resistance genes can transfer between bacteria via plasmid conjugation. Alongside drug resistance, *K. pneumoniae* can form biofilms on hospital surfaces, at infection sites and on implanted devices. Biofilms are naturally protected and can be inherently more tolerant to antimicrobials than their free-floating counterparts. There have been indications that plasmid transfer may be more likely in biofilm populations, thus creating a conjugation “hotspot”. However, there is no clear consensus on the effect of the biofilm lifestyle on plasmid transfer. Therefore, we aimed to explore the transfer of a plasmid in planktonic and biofilm conditions, and the impact of plasmid acquisition on a new bacterial host. Our data show transfer of a resistance plasmid is increased in a biofilm, which may be a significant contributing factor to the rapid dissemination of resistance plasmids in *K. pneumoniae*.

Antimicrobial resistance (AMR) is a significant global health threat. The World Health Organization lists priority pathogens for which new treatments are needed, including multi-drug resistant (MDR) and carbapenemase-producing *Klebsiella pneumoniae*, which causes substantial disease burden (1, 2). *K. pneumoniae* and members of its species complex cause infections at various body sites, including the urinary tract, the respiratory tract, and the bloodstream (3). Amongst the global high-risk MDR *K. pneumoniae* clones are those belonging to clonal group 15 (CG15), which includes sequence type (ST) 14 *K*. *pneumoniae* (4). MDR ST14 isolates have been frequently isolated and reported on, including in studies from India, the Arabian Peninsula, the UK, and the USA (5 - 8). ST14 was often associated with *bla*_NDM_ and *bla*_OXA-48-like_ carbapenemases (5, 7 - 9), and some ST14 isolates were pan-drug resistant (8). A large study in the UK looked at resistance to cefipime/zidebactam, and found that the strains resistant to this new therapy were disproportionate from ST14 (7). A recent study of hypervirulent (hv) *K. pneumoniae* found that ST14 was the most common ST identified from infected neonates, and included both carbapenem sensitive and resistant isolates (10).

In the European Economic Area, the number of infections and deaths caused by carbapenem-resistant *K. pneumoniae* (CRKP) has increased more than for any other bacterial infection (11). Carbapenem resistance is due primarily to the action of carbapenemase enzymes, the genes for which are frequently located in plasmids. One key example is the *bla*_NDM_ gene family. These genes encode metallo-beta-lactamases and have become globally distributed (12, 13). In addition to carbapenem resistance, *K. pneumoniae* often carry additional antibiotic resistance genes (ARGs), many of which are also plasmid-encoded. For example, Gorrie et al. (3) found the majority (86%) of *K. pneumoniae* isolates contained plasmids (3), which is problematic because plasmids can potentially transfer to new hosts via a process termed conjugation, providing a route of ARGs transmission and contributing to the spread of AMR (4, 14, 15). During conjugation, plasmid DNA is transferred from a donor to a recipient via a pilus which links the donor and recipient cells (16 - 18). The efficiency of conjugation can be enhanced by mating pair stabilization, where interactions between the plasmid-encoded outer-membrane protein TraN on the donor cell interact with outer-membrane proteins on the recipient cell, such as OmpK36 (19). A recent study of CRKP in Europe found that successful dissemination of carbapenem resistance could occur through success of a specific clone, a specific plasmid, or transient association of a strain with different plasmids (13).

As the conjugation process requires cell-cell contact, there has been interest in the potential interplay between conjugation and biofilms. Biofilms consist of aggregated cells which are encased in a matrix and are usually attached to a surface (20, 21). In nature, most bacteria are thought to live in biofilms, and *K. pneumoniae* often exists in this lifestyle (22 - 24). Biofilms are clinically relevant as they form on hospital surfaces and at infection sites, and can include *K. pneumoniae* (25 - 28). Hospital settings could thus provide an optimal environment for ARGs transfer, with the combination of biofilms, antibiotic pressure, and plasmid-encoded ARGs.

Although higher conjugation frequencies have been reported in biofilms compared to in planktonic populations, there remains a lack of consensus as to the relationship between biofilms and conjugation (22, 29, 30). There is also some evidence that the conjugative pili encoded by some plasmids themselves promote adherence, leading to increased biofilm formation (31). Taken together, these factors hint at a putative positive feedback loop between biofilm formation, plasmid carriage, and plasmid transfer. Therefore, this study provides additional data on the interplay between biofilms and conjugation in a clinical *K. pneumoniae* isolate.

Due to the prevalence of AMR plasmids in hospital-associated bacteria such as *K. pneumoniae*, the presence of biofilms in hospital settings, and the importance of investigating plasmid transfer from clinically-relevant strains, we hypothesized that the biofilm lifestyle would improve conjugation of an AMR plasmid from a recent *K. pneumoniae* patient isolate. We also hypothesized that plasmid acquisition would have a distinct impact upon bacterial cells grown in biofilms or planktonically.

## RESULTS

### MDR *K. pneumoniae* clinical isolate with plasmid-borne *bla*_NDM-1_

A CRKP strain isolated from a urine sample taken at the Queen Elizabeth Hospital (Birmingham, UK) was used for this study, and named “CPE16.” A combined long- and short-read sequencing approach (Oxford Nanopore and Illumina) was used to characterize the strain and determine its plasmid content. CPE16 was classified as ST14 using Multilocus Sequence Typing (MLST), and a core-genome phylogeny comparing this isolate to publicly available *K. pneumoniae* species complex sequences revealed CPE16 falls within the *K. pneumoniae sensu stricto* group (Fig. S1).

The complete CPE16 genome consists of its chromosome and four plasmids. The large plasmids pCPE16_2 and pCPE16_3 are H- and F-types, respectively, and pCPE16_4 is a ColE1-like small plasmid (Table 1). The smallest plasmid, pCPE16_5, was not typed by PlasmidFinder, but we found that it encodes a replication initiation protein related to that of phage IKe. Thus, we conclude that like IKe (32), pCPE16_5 utilizes rolling-circle replication.

**TABLE 1:** Complete CPE16 genome characteristics

Contig#, molecule	Size (bp)	Replicon type^*a*^	Antibiotic resistance genes
1, chromosome	5,293,517	–	*bla*_SHV-106_, *mdf(A*), *fosA6, oqxAB, bla*_OXA-1_, *catB3, aacA4, dfrA1*, *sat2*
2, pCPE16_2	248,840	H-type^*b*^	*msr(E)-mph(E*), *armA*, *sul1*, *aadA1*, *aadA2*, *dfrA12*
3, pCPE16_3	119,165	FII(K)-2:FIB^*c*^	*bla*_NDM-1_, *bla*_CTX-M-15_, *bla*_TEM-1B_, *bla*_OXA-9_ ^*d*^, *ble*_MBL_ *, aadA1, aacC4, qnrS1, aphA6*
4, pCPE16_4	4,173	θ-RNA^*f*^	–
5, pCPE16_5	2,095	Rolling-circle^*e*^	–

^*a*^ Replicon types determined using a combination of PlasmidFinder and PubMLST output, or through comparison to replicons of known types.

^*b*^ Contains replicons identical to the PlasmidFinder references for pNDM-Mar (GenBank accession JN420336).

^*c*^ FII(K) replicon sub-typed using PubMLST. The FIB replicon is identical to that of the FIB (pQil) reference (GenBank accession JN233705 ) in PlasmidFinder but could not be sub-typed by the PubMLST database.

^*d*^ Produces a non-functional OXA-9 due to the introduction of a premature stop codon by a single nucleotide polymorphism.

^*e*^ Not typed by PlasmidFinder. Contains a gene for a putative rolling-circle replication initiation protein.

^*f*^ Identified as Col440I by PlasmidFinder, this is a theta-replicating, RNA-initiating plasmid similar to ColE1.

**Fig 1:**
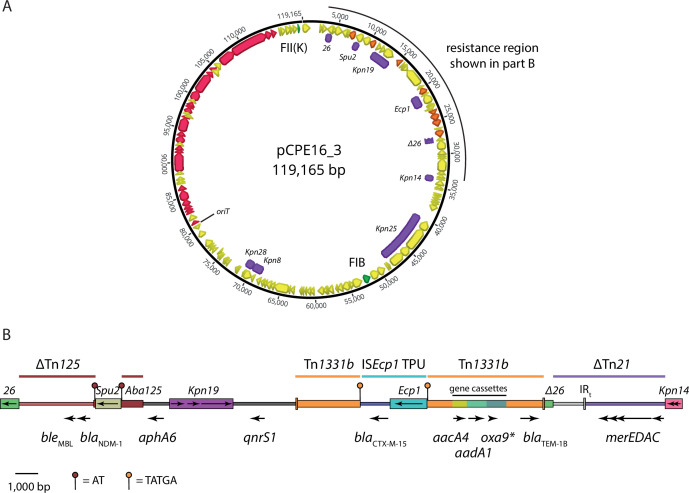
(A) Multi-drug resistance plasmid pCPE16_3. Orange arrows indicate AMR genes, green arrows indicate replication genes (labeled FII(K) and FIB), pink arrows indicate conjugation module genes, and yellow arrows indicate other coding sequences (CDS) identified by Prokka. Insertion sequences (IS) are shown as labeled purple boxes. All essential conjugation module genes (35) were present on the basis of manual annotation using plasmid *F* (GenBank accession AP001918) as a reference. Maps were prepared using Geneious Prime 2021.1 (B) Scaled diagram of the 30.3 kbp region that contains the antibiotic resistance genes in pCPE16_3. IS are shown as colored boxes, with names labeled above and the orientations of transposase genes indicated by arrows inside. The locations and orientations of antibiotic resistance genes are shown by labeled arrows below the diagram. The extent of sequences derived from complete, partial (∆) or interrupted transposons, and TPUs are indicated by labeled colored lines above the diagram. The positions and sequences of target site duplications generated by insertion of IS*Spu2* and the IS*Ecp1* TPU are indicated as outlined in the key below. AMR, antimicrobial resistance; TPU, transposition unit.

The CPE16 genome contains multiple antibiotic resistance genes, including the carbapenemase gene *bla*_NDM-1_ (Table 1). Along with additional beta-lactamase genes (*bla*_CTX-M-15_ ***,*** *bla*_TEM-1B_, and a non-functional *bla*_OXA-9_), a quinolone resistance gene (*qnrS*), aminoglycoside resistance genes (*aadA1*, *aacC4*, and *aphA6*), and a bleomycin resistance gene (*ble*_MBL_)· *bla*_NDM-1_ were found in the 119 kbp F-type plasmid pCPE16_3. The pCPE16_3 backbone includes FII(K) and FIB replicons, as well as a complete and uninterrupted F-like conjugation module (33), spanning approximately 34 kbp between *finO* and *traM* (Fig. 1A). The antibiotic resistance genes in pCPE16_3 were located in a complex 30 kbp resistance region, which contained multiple complete or partial translocatable elements (Fig. 1A). The *bla*_NDM-1_ and *ble*_MBL_ genes, derived from Tn*125* (34), were flanked by a copy of IS*26* and an IS*Aba125* interrupted by an IS*Sup2*-like element (Fig. 1B). The *aphA6* and *qnrS1* genes were either side of IS*Kpn19*, and the remaining resistance genes were in a Tn*1331b* element (GenBank accession GU553923) that had been interrupted by insertion of a 2,971 bp IS*Ecp1-bla*_CTX-M-15_ transposition unit (Fig. 1B).

### pCPE16_3 transfers at a high frequency in planktonic culture

Since its sequence suggested that pCPE16_3 was conjugative, we wanted to confirm this prediction in planktonic liquid cultures. However, to enable conjugation assays, a suitable recipient strain was needed which contained a unique resistance marker to distinguish it from the MDR CPE16 (Table 2). Homologous recombination was used to insert a hygromycin resistance gene (*hph*) into the chromosome of *K. pneumoniae* ATCC 43816 (ST493), disrupting the chromosomal *bla*_SHV_. PCR and sequencing were used to confirm the successful insertion event and loss of the recombineering plasmid. The *K. pneumoniae* ATCC 43816 (KP1) with *bla*_SHV_::*hph* was called KP20. Insertion of the hygromycin resistance cassette had no impact on strain growth rates [maximum growth rate in lysogeny broth (LB) for KP1: 0.0257 min^−1^ ± 0.00103, for KP20: 0.0263 min^−1^ ± 0.000479 with *P =* 0.39], and resulted in an MIC for hygromycin of >1,024 mg/L.

**TABLE 2:** Minimum inhibitory concentration data for *K. pneumoniae* CPE16

Antimicrobial/compound	CPE16 MIC^*b*^ (mg/L)	EUCAST^*b*^ breakpoint (mg/L)
Aztreonam	>32	4
Benzalkonium chloride	64	N/A^*a*^
Carbenicillin	>1,024	N/A
Cefotaxime	>256	>2
Chloramphenicol	256	>8
Ciprofloxacin	128	>0.5
Clindamycin hydrochloride	>256	N/A
Crystal violet	16	N/A
Doripenem	>1,024	>2
Erythromycin	512	N/A
Ethidium bromide	>1,024	N/A
Fusidic acid	512	N/A
Gentamicin	1,024	>2
Hygromycin	32	N/A
Meropenem	64	>8
Methylene blue	>1,024	N/A
Moxifloxacin	>32	>0.25
Nalidixic acid	>1,024	N/A
Novobiocin	512	N/A
Rhodamine 6G	>1,024	N/A
Rifampicin	32	N/A
Tetracycline	8	N/A
Ticarcillin	>1,024	>16

^*a*^ N/A, not applicable.

^*b*^ MIC, minimum inhibitory concentration; EUCAST, European Committee on Antimicrobial Susceptibility Testing.

Conjugation assays between the CPE16 donor and the KP20 recipient were performed. The number of donors and recipients was calculated at 0 and 20 h, and the number of transconjugants (TCs) was calculated at 20 h. Under these conditions, the conjugation frequency of pCPE16_3 was 6.2 × 10^−5^ (± 2.8 × 10^−5^). Fig. S2A shows the total bacterial counts of CPE16 donors, KP20 recipients, and TCs at 0 and 20 h. Raw data can be found in Data set S1; Table 1. Putative transconjugant colonies were randomly selected and PCR analysis confirmed the presence of pCPE16_3 in the KP20 recipient. Over the 20-h incubation period the donor to recipient ratio changed substantially from 1:10 to 7:1 (Fig. S3). The conjugation assays confirmed that pCPE16_3 was conjugative, and conferred resistance to the carbapenem doripenem (MIC of doripenem for KP20 was 0.016 mg/L, while for KP20/pCPE16_3 it was >16 mg/L). Other antibiotic resistance genes are present in pCPE16_3, but were not tested as part of this study.

In addition to PCR, the genomes of five transconjugants were sequenced using Illumina technology. Reads were aligned to the donor genome to determine which plasmid replicon(s) were present in the transconjugants. Read alignments confirmed that all colonies contained pCPE16_3. Interestingly, 3/5 colonies also contained the ColE1-like plasmid pCPE16_4. Examination of the pCPE16_4 sequence revealed that it does not encode a relaxase protein, and therefore likely utilizes relaxase—*in trans* mobilization (36). Consistent with this, we found that pCPE16_4 contains a 102 bp sequence that is 79% identical to the *oriT* region of pCPE16_3 (Fig. S4). We conclude that pCPE16_4 was likely mobilized by pCPE16_3 in these experiments.

### Biofilm lifestyle promotes pCPE16_3 conjugative transfer

**Fig 2:**
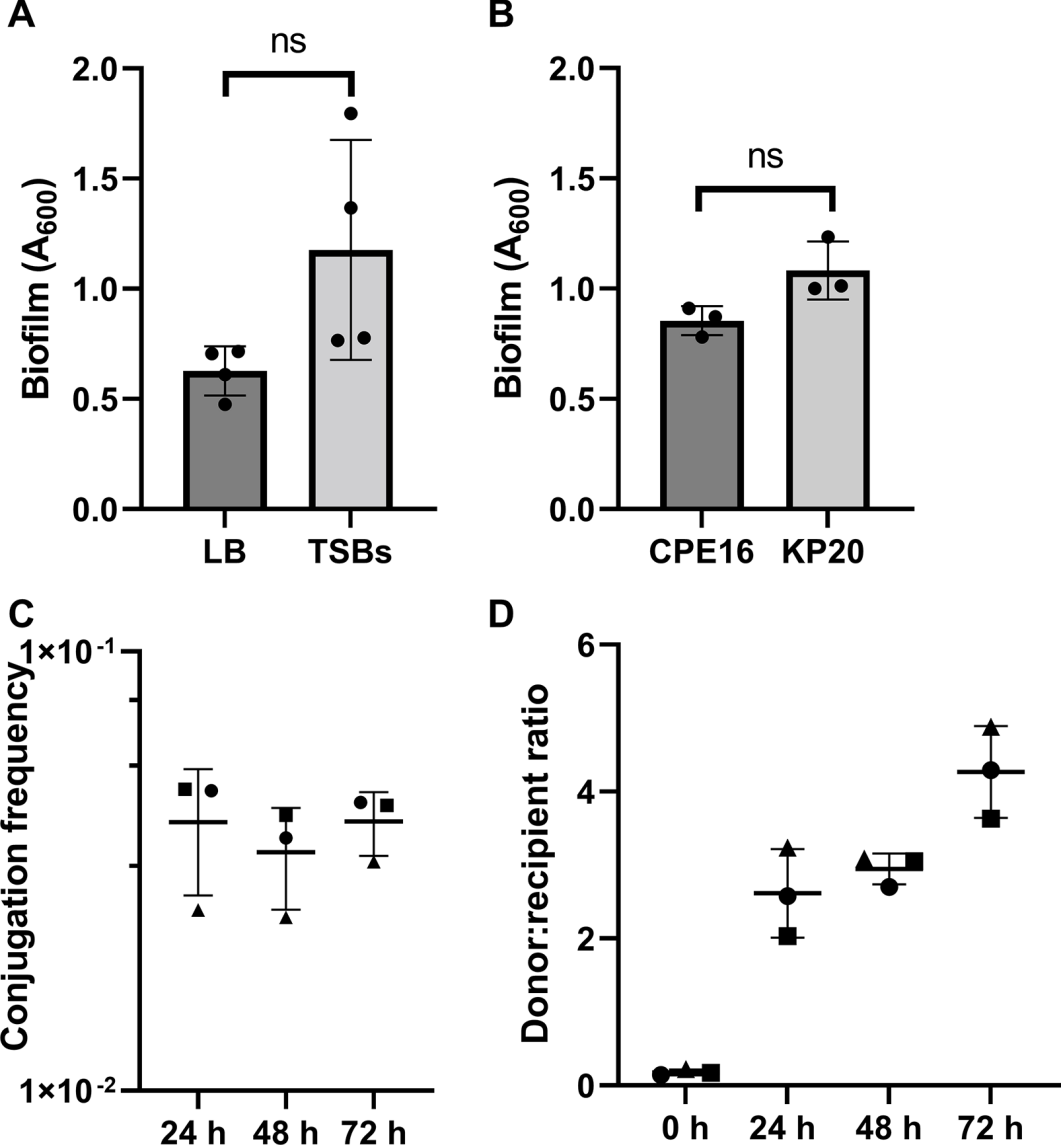
CPE16 biofilm and conjugation. (A) Mean biofilm formation (crystal violet staining) at 72 h in TSB and LB. *n* = four experimental replicates, each the mean of three biological replicates. Each biological replicate is the mean of three technical replicates. Media-only values have been subtracted. Data available in Data Set S1, Tab 2. (B) Mean biofilm formation of CPE16 and KP20 at 24 h in TSB (crystal violet staining) (*P* = 0.077, unpaired *t*-test with Welch’s correction). *n* = three experimental replicates, each the mean of three biological replicates. Each biological replicate is the mean of three technical replicates. Media-only values have been subtracted. Data available in Data Set S1, Tab 3. (C) Mean conjugation frequencies of pCPE16_3 from the CPE16 donor into the KP20 recipient in a biofilm over time. Point shape describes the experimental replicate. One-way ANOVA indicated no difference in the conjugation frequencies across the time-points (*P* = 0.71). Data available in Data Set S1, Tab 4. (D) Mean donor:recipient ratios (CPE16:KP20) over time in biofilm conjugation assays. For both (C) and (D), *n* = three experimental replicates, each the mean of four biological replicates. For all, error bars represent standard deviation from the mean. “ns” indicates “not significant”. Data available in Data Set S1, Tab 5. ANOVA, analysis of variance; LB, lysogeny broth; TSB, tryptic soy broth.

We anticipated that the close contacts afforded by the biofilm lifestyle would promote horizontal gene transfer (HGT) events. To test this, we first wanted to establish a suitable biofilm model. As Cusumano et al. (37) suggest biofilm production for *K. pneumoniae* is facilitated in supplemented tryptic soy broth (TSB) media, we compared biofilm formation in TSB versus LB. Whilst the difference was not statistically significant, there was a clear trend of improved biofilm formation using TSB (Fig. 2A); therefore, this medium was selected for our model. Next, we evaluated biofilm formation of the CPE16 donor and KP20 recipient at 24 h, and found no statistically significant difference between biofilm formation of the two strains (Fig. 2B).

Conjugation frequency of pCPE16_3 in the biofilm was measured at 24, 48, and 72 h. The data show high levels of conjugation (±standard deviation) at all three time points compared to in planktonic populations: 4.1 × 10^−2^ (±1.3 × 10^−2^) at 24 h, 3.5 × 10^−2^ (±9.1 × 10^−3^) at 48 h, and 4.1 × 10^−2^ (±6.8 × 10^−3^) at 72 h (Fig. 2C). The bacterial counts for donor, recipient and transconjugants at each time point are available in Fig. S2B. Over the course of the assay, the donor to recipient ratio shifted, from approximately 1:10 at the start of the experiment to 4:1 at 72 h (Fig. 2D). This change in ratio, which may be due to the donor killing the recipient for example via a Type 6 Secretion System (38), was less pronounced in the biofilm model compared to the planktonic assays, and less apparent at the 24- and 48-h time point from the biofilm experiment.

As with the planktonic conjugation assays, colonies were selected at random for PCR to confirm plasmid presence in the recipient strain and all were identified as transconjugants. Two transconjugant colonies were whole genome-sequenced. Similar to what was seen in the planktonic conjugations, both sequenced colonies contained pCPE16_3, one colony had also acquired pCPE16_4, while the other colony had acquired pCPE16_2 in addition to pCPE16_3.

### Acquired plasmids are stably maintained in KP20 and have no effect on fitness or biofilm formation

**Fig 3:**
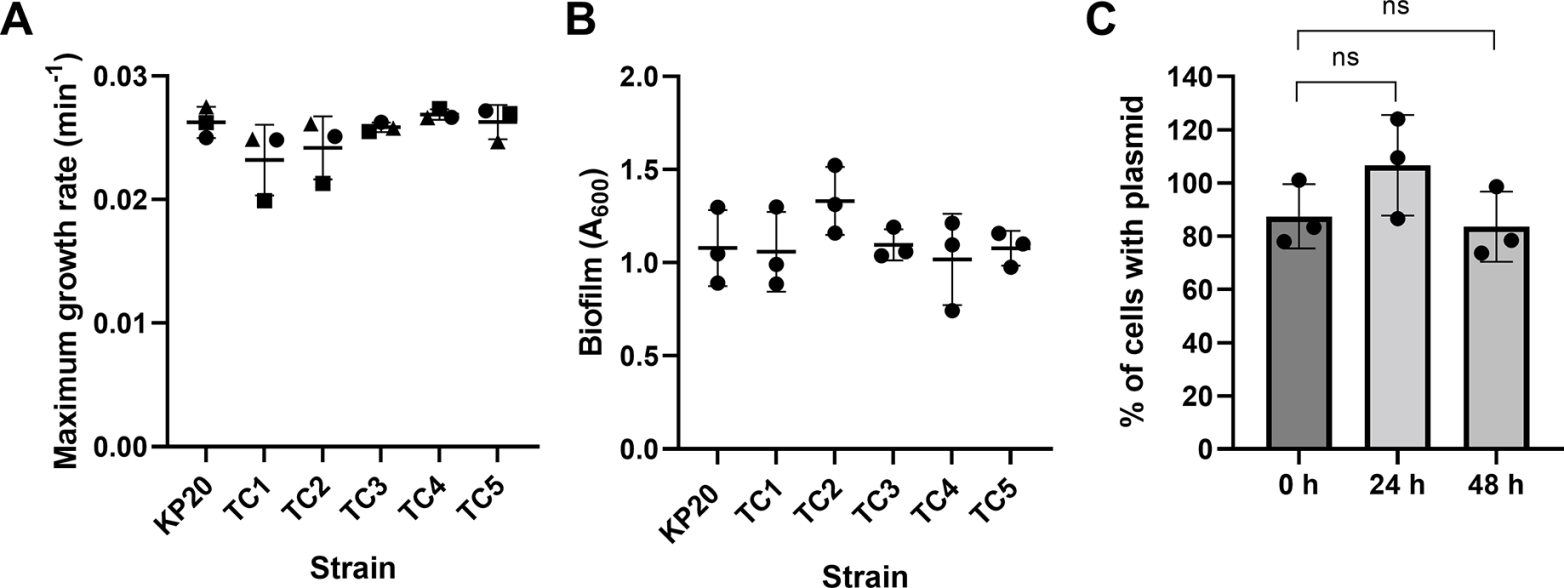
Transconjugant phenotypes. (A) Maximum growth rates for KP20 recipient strain and KP20/pCPE16 transconjugants 1-5 (TC1-5). Data available in Data Set S1, Tab 6. (B) Biofilm formation as determined by crystal violet assay at 72 h for KP20 and KP20/pCPE16 TC1-5. Data available in Data Set S1, Tab 7. (C) Persistence of pCPE16_3 in KP20 over time, displayed as the percentage of cells containing the plasmid. One-way ANOVA indicated no difference when comparing maximum growth rates of KP20 to transconjugants or when evaluating biofilm formation. Mann-Whitney *U* test indicated no significant difference between plasmid prevalence at 24 or 48 h. *N* = three experimental replicates, each comprising three biological replicates. For growth rates and biofilm assay, biological replicates were determined from three technical replicates. Data available in Data Set S1, Tab 8. ANOVA, analysis of variance.

Fitness and biofilm formation were assessed using the five sequenced KP20/pCPE16_3 transconjugants from the planktonic conjugation assays. Of these, transconjugant (TC) 1-3 contained both pCPE16_3 and pCPE16_4, while TC4 and TC5 contained only pCPE16_3. None of the plasmids had a statistically significant impact upon maximum growth rate or biofilm formation (Fig. 3A and B). In addition, the persistence of pCPE16_3 in KP20 was monitored over 48 h in the absence of selection, and no statistically significant plasmid loss was observed (Fig. 3C).

### Impact of plasmid carriage on chromosomal gene expression is most pronounced in biofilm and 24 h planktonic lifestyles

To determine the impact of plasmid carriage and/or biofilm formation on gene expression, RNA sequencing was carried out on KP20 ± pCPE16_3 to compare the transcriptome across three “lifestyles”: planktonic exponential growth phase, planktonic stationary phase (24 h), and biofilm (24 h). For technical reasons, only two biological replicates were included for the planktonic stationary phase analysis, while all others had three biological replicates. From the data we examined two main questions: (i) what is the impact of plasmid carriage on KP20 chromosomal gene expression in each lifestyle? and (ii) what is the impact of lifestyle on plasmid gene expression?

**Fig 4:**
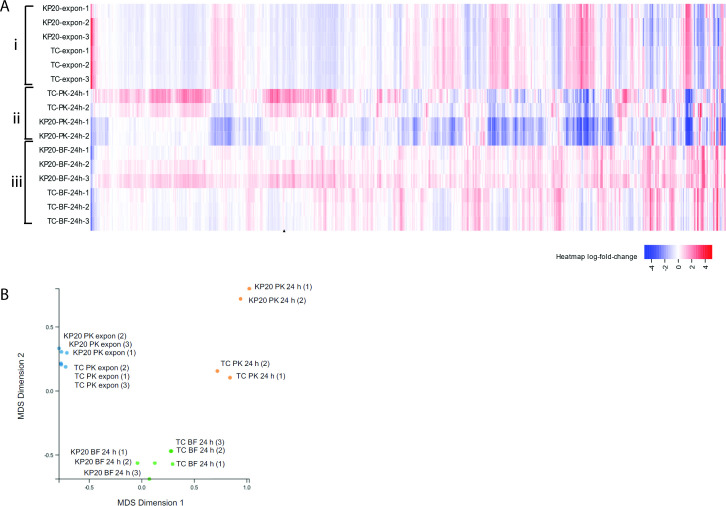
(A) Differential expression of chromosomal genes across (i) planktonic exponential indicated by “-expon-”, (ii) planktonic 24 h (“−PK-24 h−”), and (iii) 24 h biofilm (“−BF 24 h−”) conditions from Degust v4.2-dev . Each colored box in the vertical direction corresponds to a single sample within A, B, and C conditions. Each box in the horizontal direction corresponds to an individual gene. Columns ordered as per the Degust default settings: following a variance stabilizing transform, an algorithm clusters genes which have similar log_2_ fold change values across the samples. Color coding relates to the average log_2_ fold change. No thresholds have been applied. Three biological replicates each of the wild type (WT) KP20 and TC strains were compared for each condition except for the planktonic 24-h condition where two biological replicates were included. (B) MDS plot from Degust v4.2-dev of similarity between samples in lifestyle groups compared to the KP20 reference genome. All samples, grouped by lifestyle, are compared to all other samples. Biological replicates of the WT KP20 and TC KP20/pCPE16_3 are displayed as individual points for planktonic exponential (blue), planktonic 24 h (orange), and biofilm 24 h (green) groups. Data available in Data Set S1, Tab 9. MDS, multi-dimensional scaling; TC, transconjugant.

The data show that plasmid presence had relatively little impact on chromosomal gene expression in exponential planktonic populations but had a more pronounced impact in 24-h planktonic and biofilm populations (Fig. 4A). It was also evident that lifestyle had a much greater impact on chromosomal gene expression than plasmid presence. When evaluated using multi-dimensional scaling (MDS) plots, samples from the exponential and biofilm conditions clustered well in their sample groups regardless of plasmid carriage, while the planktonic 24-h samples clustered based on plasmid carriage. Overall, samples from each lifestyle condition displayed distinct gene expression patterns, indicated by their clustering as separate groups (Fig. 4B).

**Fig 5:**
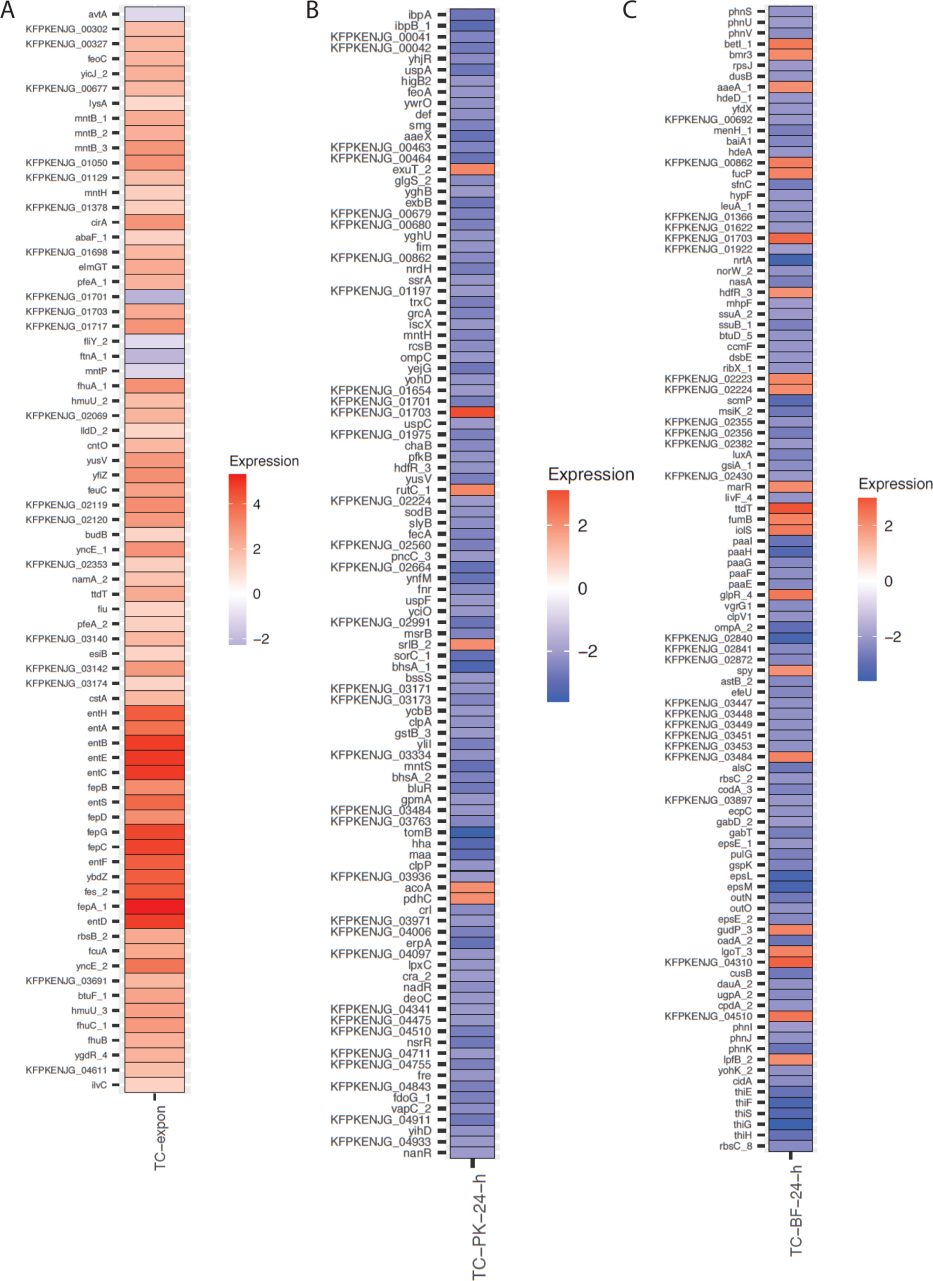
Statistically significant differential expression of chromosomal genes in genome order. (A) Planktonic exponential condition: comparison of KP20 to the transconjugant (TC-expon) where the adjusted *P* value <0.05 and log_2_ fold change mean expression was between ≥1and ≤−1. Data available in Data Set S1, Tab 10. (**B**) Planktonic 24 h condition: comparison of KP20 to the transconjugant (TC-PK-24-h) where the adjusted *P* value <0.05 and log_2_ fold change mean expression was between ≥2 and ≤−2. Data available in Data Set S1, Tab 11. (C) Biofilm (BF) 24 h condition: comparison of KP20 to the transconjugant (TC-BF-24-h) where the adjusted *P* value <0.05 and log_2_ fold change mean expression was between ≥2 and ≤−2. For all plots, locus tags represent hypothetical proteins. Plots were prepared usidng ggplot2. Data available in Data Set S1, Tab 12. TC, transconjugant.

In the planktonic exponential lifestyle, comparison of KP20 with KP20/pCPE16_3 indicated a total of 73 genes were significantly differentially expressed, with higher expression mostly observed in the transconjugant (68/73, 93%) (Fig. 5A). Clusters of orthologous genes (COG) categories (39) were used to assess these categories which included inorganic ion transport and metabolism, secondary metabolites, and transcription (Fig. S5A). Of the genes to which functions could be assigned, 33% (24/73) were predicted to have a role in iron binding, capture, uptake, and transport (Fig. 5A). The downregulated genes were involved in amino acid metabolism (*avtA*), iron storage (*ftnA*), L-cystine-binding (*fliY*), and manganese efflux (*mntP*).

In contrast to the relatively small number of genes with altered expression in the exponential phase, in the planktonic 24 h and biofilm conditions, 911 and 925 genes, respectively, were differentially expressed in the KP20/pCPE16_3 transconjugant. COG categories were used to assess the genes with altered expression as a result of plasmid carriage. In the planktonic 24-h condition the profile of COG categories was highly varied. These included a relatively even split of upregulation (54.5%) and downregulation (45.5%), with a higher proportion of upregulated genes overall, specifically across several transport and metabolism categories, alongside transcription and energy production and conversion (Fig. S5B). In the biofilm, there was upregulation of transcription-associated genes, and downregulation of genes involved in metabolism, energy production and conversion, and inorganic ion transport and metabolism (Fig. S5C).

To investigate the 24-h planktonic and biofilm data in greater detail, a more stringent cut off of log_2_ fold change of between ≤−2 and ≥2 was applied. For the planktonic condition, this cut-off criteria resulted in a list of 104 genes, of which 98/104 (94%) were downregulated (Fig. 5B). Although many (23) genes were annotated as hypothetical proteins, downregulated genes included those involved in stress response modulation and metal transport, amongst others. Additionally, *ompK36* [homologue of *Escherichia coli ompC* (40)] encoding the outer membrane porin OmpK36 was downregulated. OmpK36 has recently been identified as a receptor for the TraN component of the conjugative pilus of the F-type plasmid pKpQIL (19). There were five upregulated genes meeting the threshold criteria, annotated as: *acoA* (oxidoreductase sub-unit), *pdhC* (acetyltransferase component), *srlB* (phosphotransferase system component), *rutC* (aminoacrylate deaminase), *exuT* (hexuronate transporter), and a hypothetical protein.

For the biofilm condition, the more stringent cut-off criteria resulted in a list of 107 genes, of which 85/107 (79%) were downregulated (Fig. 5C). Downregulated genes in the biofilm condition included those with diverse functions, involved in processes such as translation, management of acid stress, and secretion. There was also downregulation of *ompK35* [homologue of *E. coli ompF* (40)]. Four upregulated genes were annotated as “transcriptional regulators”: *hdfR, betl, glpR,* and *marR,* with *marR* encoding the “multiple antibiotic resistance” regulator protein. Additionally, *aaeA*, encoding an efflux pump sub-unit, was also upregulated during growth in biofilm. Interestingly, comparison of the chromosomal genes with altered expression between the three different lifestyles revealed that only six common genes had altered expression in two or more lifestyles upon plasmid acquisition (adjusted *P* value <0.05 and log_2_ fold change mean expression between ≥2 and ≤−2) (Table S1).

### Lifestyle impacts expression of plasmid genes

**Fig 6:**
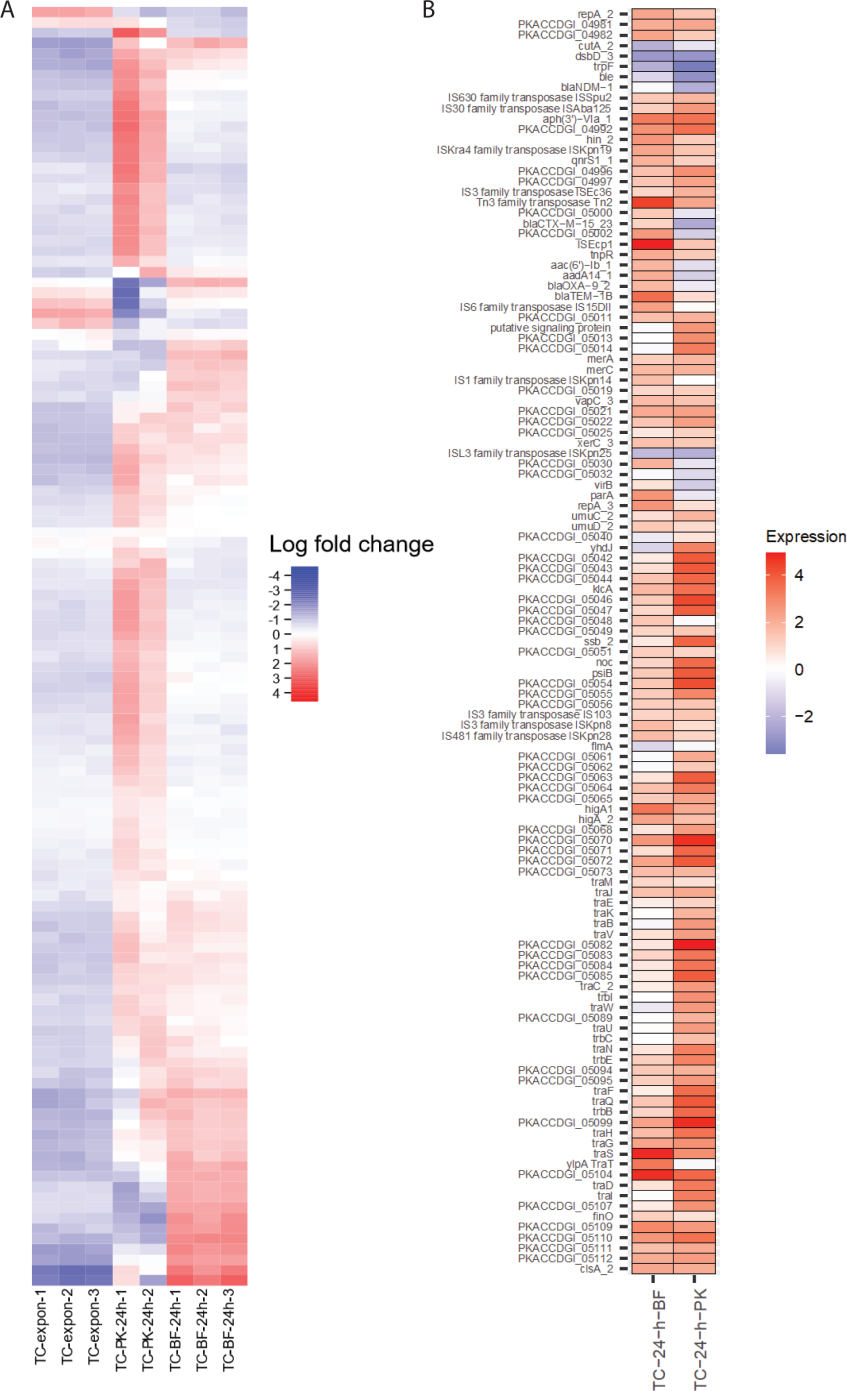
(A) Differential expression of 123 plasmid genes across planktonic exponential (“-expon-”), planktonic 24 h (“-PK-24 h-”), and 24 h biofilm (“-BF 24 h-”) conditions from Degust v4.2-dev. Each colored box in the vertical direction corresponds to a single sample. Each box in the horizontal direction corresponds to an individual gene. Columns ordered as per the Degust default settings: following a variance stabilizing transform, an algorithm clusters genes which have similar log_2_ fold change values across the samples. Color coding relates to the average log_2_ fold change. No thresholds have been applied. Three biological replicates each of the WT (KP20) and transconjugant (TC) strains were compared for each condition except for the planktonic 24 h condition where two biological replicates were included. Data available in Data set S1, Tab 13. (B) Statistically significant differential expression of plasmid genes relative to the planktonic exponential condition in plasmid genome order: a gene is only shown if mean differential gene expression was significantly different (adjusted *P* value <0.05, log_2_ fold change of between ≥1 and ≤−1) between the planktonic exponential phase and at least one of the comparator conditions (biofilm 24 h and planktonic 24 h). Plot was prepared using ggplot2. Data available in Data Set S1, Tab 14.

Next, we examined plasmid gene expression in the three different lifestyles. MDS plots including both chromosomal and plasmid sequence data produced similar patterns to the chromosome-only MDS plots, where lifestyle-dependent clustering was observed (Fig. S6). Investigating the data in more detail suggests each lifestyle had a distinct impact on plasmid gene expression (adjusted *P* value <0.05, log_2_ fold change set to between >1 and <−1) (Fig. 6A). Broadly speaking, plasmid gene expression was downregulated in the exponential phase, and there were distinct up/downregulation patterns for planktonic 24 h and biofilm lifestyles (Fig. 6A). Differentially expressed plasmid genes were assessed relative to the planktonic exponential condition (Fig. 6B). The data indicated many plasmid genes were upregulated in both the planktonic stationary phase and biofilm samples (60/123, 49%). For the lifestyles individually, 94/106 (89%) differentially expressed genes were upregulated in the planktonic stationary versus the exponential phase, and 76/82 (93%) differentially expressed genes were upregulated in the biofilm versus the planktonic exponential condition.

Of the plasmid genes with a function assigned (71/123, 58%), 15/71 (21%) encode proteins implicated in mobile element transposition, such as transposases. In most cases (10/15, 67%) these transposition genes were upregulated in both the stationary and biofilm conditions (adjusted *P* value <0.05, log_2_ fold change set to between >1 and <−1). Nine plasmid genes encoding antimicrobial resistance proteins were identified, 2/9 (*aphA6* and *qnrS1*) were upregulated in both the stationary and biofilm conditions compared to the exponential condition and 5/9 (*bla*_CTX-M-15_, *aacA4*, *aadA1*, *bla*_OXA-9_, and *bla*_TEM-1B_) were upregulated in the biofilm condition only. Interestingly, *bla*_NDM-1_ was downregulated in the 24-h planktonic condition, and largely unaffected in the biofilm. Genes involved in toxin-antitoxin systems and antirestriction were upregulated in both lifestyles. Expression of *parA* (plasmid segregation) was upregulated in the biofilm but not significantly different in the planktonic stationary phase.

Concerning the conjugation module, in the planktonic stationary condition, all of the conjugation module genes were upregulated versus the planktonic exponential condition except for *traT*, *traM,* and *finO* which did not meet the threshold for differential expression. In the biofilm condition, transcription of several conjugation module genes was unchanged versus the planktonic exponential condition, including genes involved in pilus assembly (*traE, traK)*, the *traI* helicase, *traN* mating pair stabilization protein, the *traC* ATP binding protein, and the *traD* coupling protein. Across both the planktonic 24 h and biofilm 24 h conditions, *traJ* was upregulated versus the planktonic exponential condition. TraJ is a positive regulator of the entire transfer operon in F-like systems (41). It is worth noting that for this RNA sequencing experiment, bacteria were grown in pure culture, meaning no potential recipient cells for the plasmid were present. This may have an impact on conjugation module gene expression.

## DISCUSSION

Here we have sequenced the genome of, and further characterized, a clinical urinary isolate of *K. pneumoniae* containing a 119 kbp plasmid carrying *bla*_NDM-1_, *bla*_CTX-M-15_, and *bla*_TEM-1B_ carbapenemase and beta-lactamase genes. We showed that this plasmid can be transferred efficiently by conjugation in planktonic culture, and at orders-of-magnitude higher frequencies in a biofilm. The growth kinetics for transconjugants which had acquired pCPE16_3 were indistinguishable from the plasmid-free parental strain, and carriage of pCPE16_3 had no impact on transconjugant biofilm formation. In the absence of selection, the plasmid was maintained over 48 h. Our gene expression analysis indicated that plasmid presence had a distinct impact on chromosomal gene expression in 24-h planktonic and biofilm lifestyles, and less impact in exponential growth. It is unsurprising that the different lifestyles tested had significant impacts upon chromosomal gene expression. Interestingly, these data showed that (i) in biofilm, planktonic 24 h, and to a lesser extent exponential conditions the transconjugant had distinct gene expression compared to the isogenic plasmid-free strain, indicating that the presence of the plasmid alters the way the host cell adapts to these lifestyles. (ii) Lifestyle had a distinct impact on plasmid gene expression, demonstrating that not only do bacteria alter their chromosomal gene expression as a result of environmental changes, but they also regulate the plasmid genes differently in these conditions.

Although plasmids/their accessory genes can benefit their host cell, plasmid carriage can also impose a fitness cost under some conditions. This supposed cost/benefit conflict has been termed the “plasmid paradox” (42). Many resistance plasmids carry toxin-antitoxin systems and partitioning systems which help to ensure their maintenance once established in a bacterial host. Compensatory evolution through mutation can reduce cost-of-carriage, and help to resolve specific genetic conflicts which lead to fitness costs (43 - 45). However, not all plasmids produce a fitness cost for a particular host. Some plasmids can be maintained without positive selection, and may not impact cell growth (46). Indeed, some plasmids may increase host fitness without selection (46, 47). For example, a recent study determined that carriage of the pOXA-48_K8 plasmid was of benefit for some patient gut microbiota isolates on the basis of competition experiments and growth assays (48). Another recent study found that some *E. coli* strains which acquired the pLL35 plasmid, carrying *bla*_CTX-M-15_ and *bla*_TEM-112_ beta-lactamases alongside other resistance genes, had enhanced growth versus the plasmid-free recipient (49). In work comparing two pKpQIL-like plasmids, gene expression changes rather than mutations were sufficient to reduce plasmid carriage costs (46). In line with these studies, our assays did not detect any fitness costs associated with carriage of pCPE16_3 by the *K. pneumoniae* host. The cost-benefit balance may switch quickly upon encountering new environmental conditions (50), and host background can have a large impact on fitness (48).

Biofilms are a problem in hospital environments where they can form on surfaces and are commonly found at infection sites (26, 28, 51). In contrast to biofilm cells, planktonic cells are suspended individually in liquid and are therefore likely to have equal exposure to environmental conditions (20, 23) and are unlikely to remain in close proximity to each other, reducing the probability of cell-cell contact (30). The co-evolutionary trajectories of plasmids and hosts are unique in biofilms compared to planktonic populations (52). In addition, cells in a biofilm are in close proximity to each other which may have implications for HGT permitted by cell-cell contact (30). There remains a lack of consensus on the interplay between biofilm and HGT, with some reports that biofilm promotes the process and others indicating limited plasmid transfer [reviewed by reference (30)]. In our system, the biofilm lifestyle was associated with higher levels of conjugation compared to in planktonic culture. This is also in line with previous work (53), which found plasmid transmission in a *K. pneumoniae* biofilm occurred at a very high frequency (0.5 transconjugants per donor). However, as strain-plasmid combinations are unique, it is difficult to generalize about whether the biofilm lifestyle leads to increased conjugation (due to increased and prolonged cell contacts or other factors), or if the limited cell movement in a biofilm restricts the horizontal spread of conjugative plasmids. Some data suggest a conjugative plasmid may promote biofilm formation due to conjugative pili aiding surface adhesion (31). However, this effect on biofilm formation is not always observed (29), and was also not observed in our study.

Biofilms provide vastly different conditions to those encountered by planktonic cells (21), and are inherently more drug-tolerant than planktonic cells (23). Likely due to ease of manipulation, most studies on bacterial cells have been carried out on planktonic populations (54). However, it is essential to study these two lifestyles separately as the state of biofilm-embedded cells cannot be deduced from planktonic cells (21). In fact, several studies have demonstrated characteristic transcriptional profiles for cells in the biofilm lifestyle compared to in planktonic culture (55 - 57). Guilhen et al. (55) found, when comparing gene expression in *K. pneumoniae* biofilm and planktonic cultures, that transcriptional fingerprints could be determined relating to growth stages in planktonic cultures (exponential phase versus stationary phase) and biofilms (aggregates versus 3D structures and cells dispersed from a biofilm) (55). Indeed, our data support this hypothesis, with distinct chromosomal gene expression patterns in each of the three lifestyles. Together, this demonstrates that gene expression is specifically tailored to growth stage and lifestyle. It is clear that biofilms are important and unique bacterial lifestyles which require individual study. Our study adds to previous work with the observation that plasmid presence also had distinct impacts on chromosomal gene expression across the three tested conditions.

Strikingly, plasmid gene expression in each of the three lifestyles was distinct. The downregulation of plasmid gene expression during exponential phase may be explained by cells aiming to reduce the fitness burden of plasmid carriage during rapid growth. Once in stationary phase or in a biofilm, plasmid genes were generally upregulated in our data set, including genes involved in conjugation and antibiotic resistance. Our conjugation data indicated conjugation frequencies were higher in the biofilm population, yet an increase in conjugation gene expression in comparison to 24-h planktonic cells was not observed. However, our RNA-sequencing experiment did not contain any potential recipient cells, which could explain this discrepancy.

Overall, our data highlight that conjugation in biofilms is occurring at higher levels than predicted based on planktonic data. This is of particular concern as biofilms are the dominant bacterial lifestyle in many settings including hospital environments and in some infections. We also show that bacteria modulate gene expression patterns based on lifestyle, and in our data, plasmid presence substantially altered these patterns. We also demonstrate that plasmid genes are differentially expressed in each lifestyle. Plasmids are important contributors to AMR and to virulence and furthering our understanding of how these mobile genetic elements interact with bacterial hosts in varied and relevant settings is thus of considerable importance.

## MATERIALS AND METHODS

### Routine culturing

Bacterial strains were routinely stored in glycerol at −80°C, grown in lysogeny broth (LB)/agar (LBA) (Sigma-Aldrich) at 37°C, with aeration (shaking) for liquid cultures. Supplemented tryptic soy broth (TSB) was prepared as per reference (37) with 25 mg/L calcium chloride, 12.5 mg/L magnesium sulphate, and 1.25% total glucose. For optical density (OD) measurements, overnight cultures (37°C, grown for ~16 h) were used and measurements were taken at 600 nm (OD_600_).

### Whole genome sequencing

Whole genome sequencing (WGS) was carried out by MicrobesNG (https://microbesng.com), with preparation of strains as per their recommendations. Sample preparation for Illumina and Oxford Nanopore sequencing and initial data analysis {trimming [Trimmomatic 0.30 (58)], assembly [Unicycler 0.4.0 (59)], and annotation [Prokka 1.11 (60)]} were done by MicrobesNG using their in-house scripts. Bandage (61) was used for assembly visualization. WGS data are available under BioProject no. PRJNA917544.

### KP20 hygromycin-resistant recipient strain construction

To insert a hygromycin-resistance cassette from reference (62) into *bla*_SHV_ on the chromosome of a rifampicin-resistant derivative of *K. pneumoniae* ATCC 43816 (kindly provided by Dr. Jessica Blair), we used the protocol described in reference (63), with some modifications. Recombineering primers (Table S2) with 40 bp homology to the chromosome and 20 bp homology to the hygromycin resistance cassette from pSIM18 were used for PCR amplification of the donor DNA molecule. First, pACBSCE was electroporated into ATCC 43816 Rif^R^ with subsequent electroporation of the PCR-amplified hygromycin resistance cassette. Successful transformants were selected on agar containing 300 mg/L hygromycin. To remove the recombineering plasmid, the strain was passaged without antibiotic. PCR and selective plating were used to confirm the presence and location of the resistance cassette, the antimicrobial resistance profile of the new recipient, and to check for loss of the recombineering plasmid pACBSCE. Growth kinetic analysis and whole genome sequencing were carried out by comparison to the ancestral strain.

### Planktonic conjugation assays

This method was developed based on the conjugation protocol described in reference (64). Donor and recipient cultures were grown overnight, sub-cultures were prepared in 5 mL LB (1% inoculum) and grown to an OD_600_ of ~0.5. Cultures (1 mL) were centrifuged (3 min, 4722× g) and media were replaced with TSB to correct the OD_600_ to 0.5. The donor and recipient were mixed at a 1:10 ratio alongside control single strain cultures. Cultures were diluted 1:5 in TSB and these were incubated statically at 37°C for 20 h. At 0 and 20 h, donor and recipient cells were plated to quantify viable counts. To determine background growth, donors were plated onto 300 mg/L hygromycin and recipients were plated onto 4 mg/L doripenem. Mixed populations were plated onto single antibiotics (doripenem or hygromycin) to select the donor and recipient, respectively, and determine the proportion of each strain. At 20 h, mixed strains were plated on dual antibiotic (doripenem 4 mg/L and hygromycin 300 mg/L) to select putative transconjugants. phosphate buffered saline (PBS)-only and media-only controls were included. Each experiment comprised four biological replicates, and the experiment was performed three independent times. Biological replicates were averaged to give data for each experimental replicate. Putative transconjugants were re-streaked on dual antibiotic to confirm growth. Conjugation frequencies were calculated for each experimental replicate as follows using values from assay endpoints:


$$Conjugation\;frequency=\frac{mean\;number\;of\;transconjugants}{mean\;number\;of\;donors}$$

To confirm transconjugants, single colonies were assessed by PCR and whole genome sequencing. Colony PCR was performed using REDtaq Ready Mix (Sigma) as per the manufacturer’s instructions, with 1 mM primers and corresponding annealing temperatures (Table S2). Agarose (1%) gel electrophoresis was used to visualize PCR products, and HyperlLadder 1 kb (Bioline) was used for size determination.

### Biofilm conjugation assays

Overnight cultures were OD_600_ corrected to 0.1 in TSB. Donor and recipient were mixed at a 1:10 ratio. Single donor and recipient cultures, and mixed cultures (2 mL) were added to wells of a CytoOne 6-well polystyrene plate (Starlab, UK). Plates were covered with a Breathe-Easy membrane (Diversified Biotech), lid and incubated statically at 37°C for 24–72 h. Donor, recipient and mixed cultures, and PBS and media controls were diluted and plated on agar as per the planktonic conjugation assay protocol at 0-, 24-, 48-, and 72-h time points. At the 24-, 48-, and 72-h time points, adhered cells were harvested by removing liquid culture and washing once with 2 mL sterile PBS. PBS was added to wells (1 mL) and the base and sides of each well were scraped twice using a cell scraper (VWR) to disrupt biofilm. Disruption to single cells was confirmed using strains containing constitutively expressed fluorescent proteins to aid visualization by confocal microscopy (data not shown). A selection of putative transconjugant colonies was re-streaked on dual antibiotic (doripenem 4 mg/L and hygromycin 300 mg/L) to confirm growth, and PCR (as above) was used to confirm identity. Conjugation frequencies were calculated as above.

### Crystal violet biofilm assays

Overnight cultures corrected to an OD_600_ of 0.1 were added to wells of a sterile Cellstar 96-well polystyrene u-bottom plate (not cell-culture treated, Greiner Bio-one). Plates were covered with a Breathe-Easy membrane (Diversified Biotech, Sigma-Aldrich) and sterile lid. These were incubated statically at 37°C for 24–72 h. After incubation, culture was removed, plates were washed with distilled water, and 0.1% crystal violet solution (Sigma-Aldrich) was added. Plates were incubated statically for 15 min at room temperature. Stain was removed, wells were washed in distilled water, and the stain was solubilized in 70% ethanol for 15 min at room temperature with shaking (60 rpm, Orbit LS Labnet International Inc.). The absorbance 600 nm (*A*_600_) was measured using a FLUOstar Optima plate reader (BMG Labtech) or a Spark microplate reader (Tecan). A minimum of three experimental replicates each consisting of three biological replicates (independent bacterial populations) were included, each the mean of three technical replicates (independent wells inoculated with the same bacterial population).

### Growth kinetics

Growth kinetics were performed and assessed as previously described (65). Overnight cultures were diluted 1:10,000 in LB or TSB in 96-well plate (Greiner Bio-one). *A*_600_ was recorded over 16 h using a plate reader. Data were analyzed by growth curves (absorbance plotted against time) and by calculating maximum growth rate (*μ*) for each experiment. *μ* was calculated as follows, where *t* refers to a given time point and *A* refers to the *A*_600_ at that time point:


$$\mu =\frac{\left(ln\left(At2\right)-ln\left(At1\right)\right)}{\left(t2-t1\right)}$$

### Plasmid stability assays

Overnight cultures were diluted 1:100,000 in TSB in a 96-well plate with lid, which was incubated statically at 37°C for 24 h. Sub-culturing (1:100,000) was repeated at 24 h and the plate incubated for an additional 24 h for a total of 48 h. At time points 0, 24, and 48 h, serial dilutions were used to enumerate bacteria grown on LBA alone, or LBA containing 2 mg/L doripenem. Each experiment included broth and PBS-only controls to detect any contamination. Experiments were completed three times independently, each consisting of four biological replicates.

### RNA sequencing

Four 10 mL overnight cultures/strain were prepared in TSB, and were used to set up three test conditions [planktonic exponential, planktonic stationary (24 h) and biofilm (24 h)]. TSB (100 mL) was inoculated with 1 mL of overnight culture and incubated at 37°C 150 rpm until mid-exponential phase when 1.8 mL of the culture was harvested by centrifugation (12,470× g for 90 s). Pellets were resuspended in 1.8 mL RNAlater (ThermoFisher) and incubated at room temperature for 30 min. Cells were harvested by centrifugation and stored at −80°C. These cells represented the planktonic exponential condition.

Next the planktonic stationary condition was set up following the same protocol as above but harvesting at 24 h. For the biofilm 24 h condition, the overnight cultures were OD_600_ corrected to 0.1. Culture (2 mL) was added to 6-well CytoOne (Starlab, UK) polystyrene plates, covered with a Breathe-Easy (Diversified Biotech) membrane and lid and incubated for 24 h statically at 37°C. After 24 h, culture was removed, wells were washed with 2 mL pre-warmed PBS, and 1.8 mL RNAlater was added. Cells were scraped from the base and sides of the plate and transferred to a microfuge tube. Cells in RNAlater were incubated at room temperature for 30 min before being harvested by centrifugation and transferred to −80°C for later use.

RNA extraction, sequencing and initial data analysis were performed by GeneWiz UK Ltd. At Azenta US, Inc. following their protocols, using a minimum of 10^6^ cells. Briefly, RNA was extracted using the RNeasy Plus mini kit (Qiagen). Following quantification and integrity checks, an rRNA depletion sequencing library was prepared (FastSelect rRNA 5S/16S/23S bacterial kit, Qiagen). For in-house analysis, hybrid genomes from MicrobesNG were annotated using Prokka (60), using flags to specify species (–Genus *Klebsiella*–usegenus–species *pneumoniae*), sequencing centre ID (–centre UoB), and force Genbank compliance (–compliant). Kallisto (66) was used to pseudo-align reads to both the recipient (accession CP115894.1) and transconjugant ( CP115894.1 combined with CP115709.1) reference genomes, using the Odd-ends RNAseq_Analysis.txt workflow by Dr. Steven Dunn, available at https://github.com/stevenjdunn/Odd-ends/blob/master/RNAseq_Analysis.txt. Data were visualized and compared using Degust v4.2-dev (https://degust.erc.monash.edu/) (67). An adjusted *P* value (false discovery rate) of <0.05 and a log_2_ fold change cut-off of 1 were used to define statistically significant differences between conditions. RNA-Sequencing heatmaps comparing expression against the planktonic exponential condition and COG category graphs were prepared using R.app GUI 1.70 (7735 El Capitan build) (68) employing ggplot2 (69). COG categories were assigned using Egg-nog 5.0 (70).

### Bioinformatics

Default parameters were used for all tools unless indicated otherwise. Anaconda version (v)4.11.0 (https://www.anaconda.com/) was used, employing Python v3.7.9. Annotation was done using BLAST searches (71) against reference sequences on GenBank (72) or from the ResFinder database (73). For conjugation module genes, GenBank sequence AP001918.1 (33) was used as a reference where possible as this entry contains annotations for an experimentally validated conjugation module. “Essential” conjugation module genes were defined based on reference (35). Insertion sequences were identified on the basis of high percentage nucleotide identity to ISfinder database sequences (http://www-is.biotoul.fr) (74). Plasmid maps were prepared using Geneious Prime v11.0.6+10 (64 bit) or Gene Construction Kit v4.5.1 (Textco Biosoftware, Raleigh, USA).

MLST: the PubMLST website (https://pubmlst.org/) (75) and MLST software (Torsten Seemann, https://github.com/tseemann/mlst) were used to type isolates. PlasmidFinder (76)/plasmidMLST (75) were used to type putative plasmids and ResFinder (73) to locate acquired AMR genes. PlasmidFinder and ResFinder databases were queried using ABRicate (Torsten Seeman, https://github.com/tseemann/abricate) or by using the webtools (https://cge.cbs.dtu.dk/services/PlasmidFinder/ and https://cge.cbs.dtu.dk/services/ResFinder/).

For phylogenetic tree construction, FASTA files for *K. pneumoniae* species complex strains were obtained using accession numbers from (77) with assistance from Dr. Axel Janssen. Prokka (v1.14.6) (60), Roary v3.11.2 (using the -e flag) (78) and RAxML v8.2.12 (79) were used for annotation, core gene alignment, and phylogenetic analysis, respectively. RAxML was run using the following parameters: raxmlHPC-PTHREADS-AVX -f a -p 13524 -s <core_gene_alignment.aln> -x 12534 -# 100 -m GTRGAMMA. iTOL Version 6.4.2 (80) was used for tree visualization.

Genome sequences were compared to published reference genomes for strain validation. Snippy (https://github.com/tseemann/snippy) was used to identify any single nucleotide polymorphisms between genomes and reference genomes. Read mapping was carried out using BWA-MEM (81) and SAMtools (82).

### Additional analysis

Unpaired *t*-tests or one-way analysis of variance was used to obtain *P* values, unless indicated otherwise. As standard, data were analyzed and plotted using Microscoft Excel version 16.16.9 and Graphpad Prism version 8.0.2.

## Data Availability

The data generated in this study have been submitted to the NCBI BioProject database under accession number PRJNA917544.

## SUPPLEMENTAL MATERIAL

Fig. S1 msphere.00170-23-s0001.tif Midpoint rooted maximum likelihood phylogenetic tree of core genes.

Fig. S2 msphere.00170-23-s0002.tif Total bacterial counts from conjugation assays.

Fig. S3 msphere.00170-23-s0003.tif Mean donor-recipient ratios.

Fig. S4 msphere.00170-23-s0004.eps Circular map of pCPE16_4 and alignment of *oriT* sequences.

Fig. S5 msphere.00170-23-s0005.tif Plasmid carriage effect on chromosomal gene expression.

Fig. S6 msphere.00170-23-s0006.tif Multidimensional scaling (MDS) plot.

Table S1 msphere.00170-23-s0007.docx Chromosomal genes with altered expression.

Table S2 msphere.00170-23-s0008.docx Primers.

Data Set S1 msphere.00170-23-s0009.xlsx Raw data files for individual experiments.

ASM does not own the copyrights to Supplemental Material that may be linked to, or accessed through, an article. The authors have granted ASM a non-exclusive, world-wide license to publish the Supplemental Material files. Please contact the corresponding author directly for reuse.
