# Supplementary figures and images for: Growth in a biofilm promotes conjugation of a blaNDM-1-bearing plasmid between Klebsiella pneumoniae strains
Source: mSphere. 2023 Jul 7;8(4):e00170-23. doi: 10.1128/msphere.00170-23 (PMC10449501; doi:10.1128/msphere.00170-23)

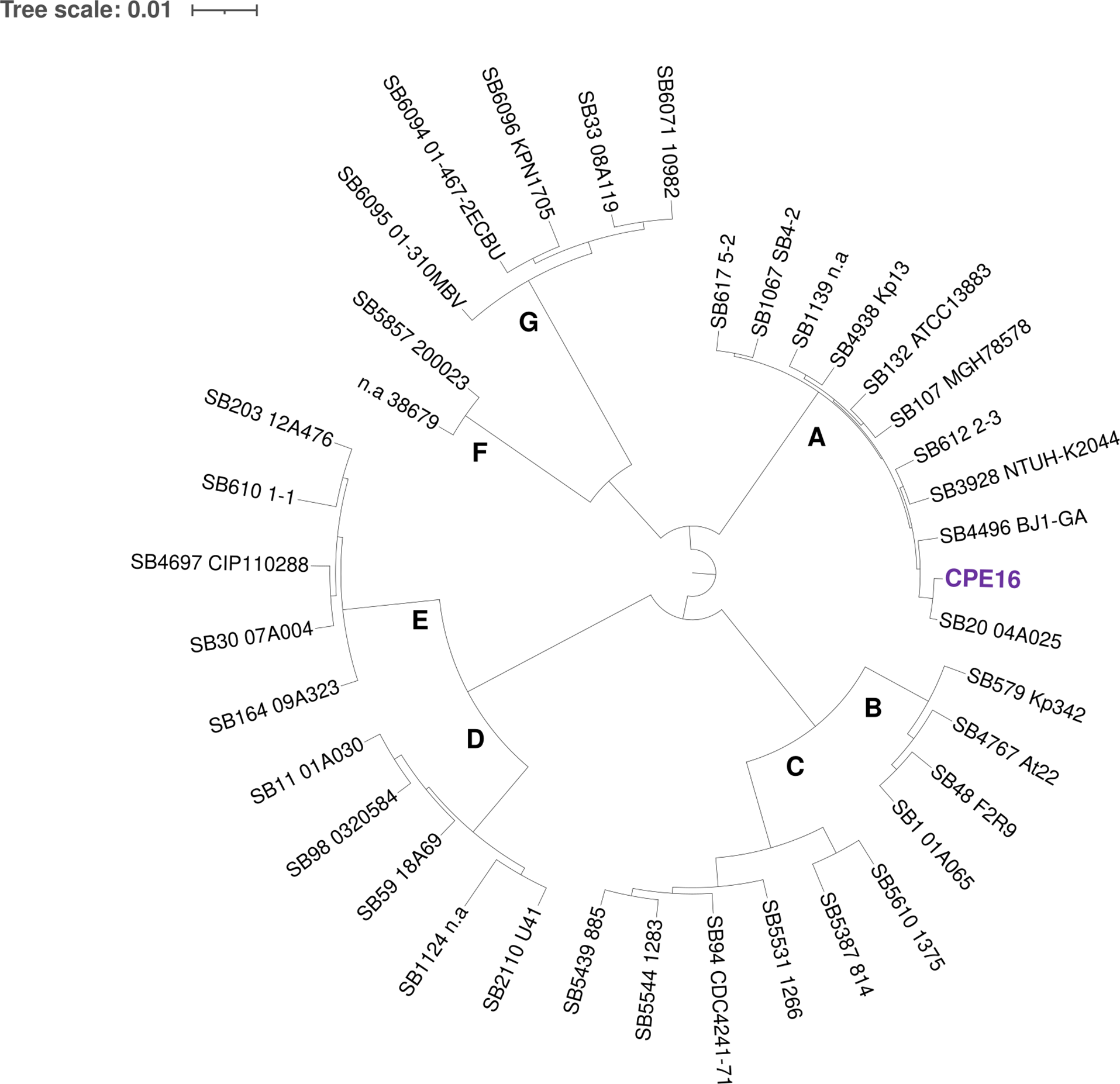

Supplement: Fig. S1 — Midpoint rooted maximum likelihood phylogenetic tree of core genes. [file msphere.00170-23-s0001.tif]

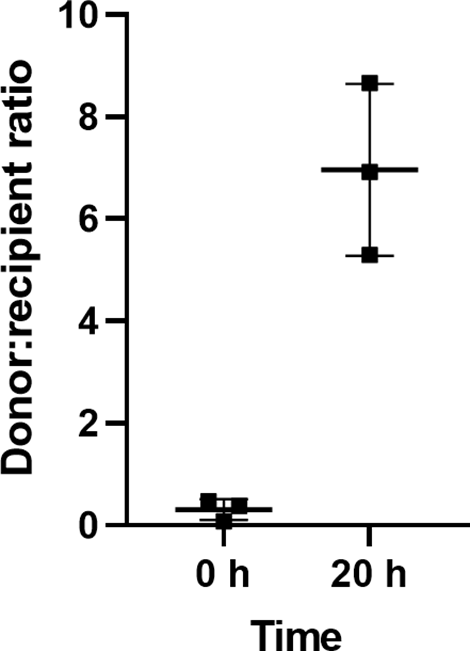

Supplement: Fig. S2 — Total bacterial counts from conjugation assays. [file msphere.00170-23-s0002.tif]

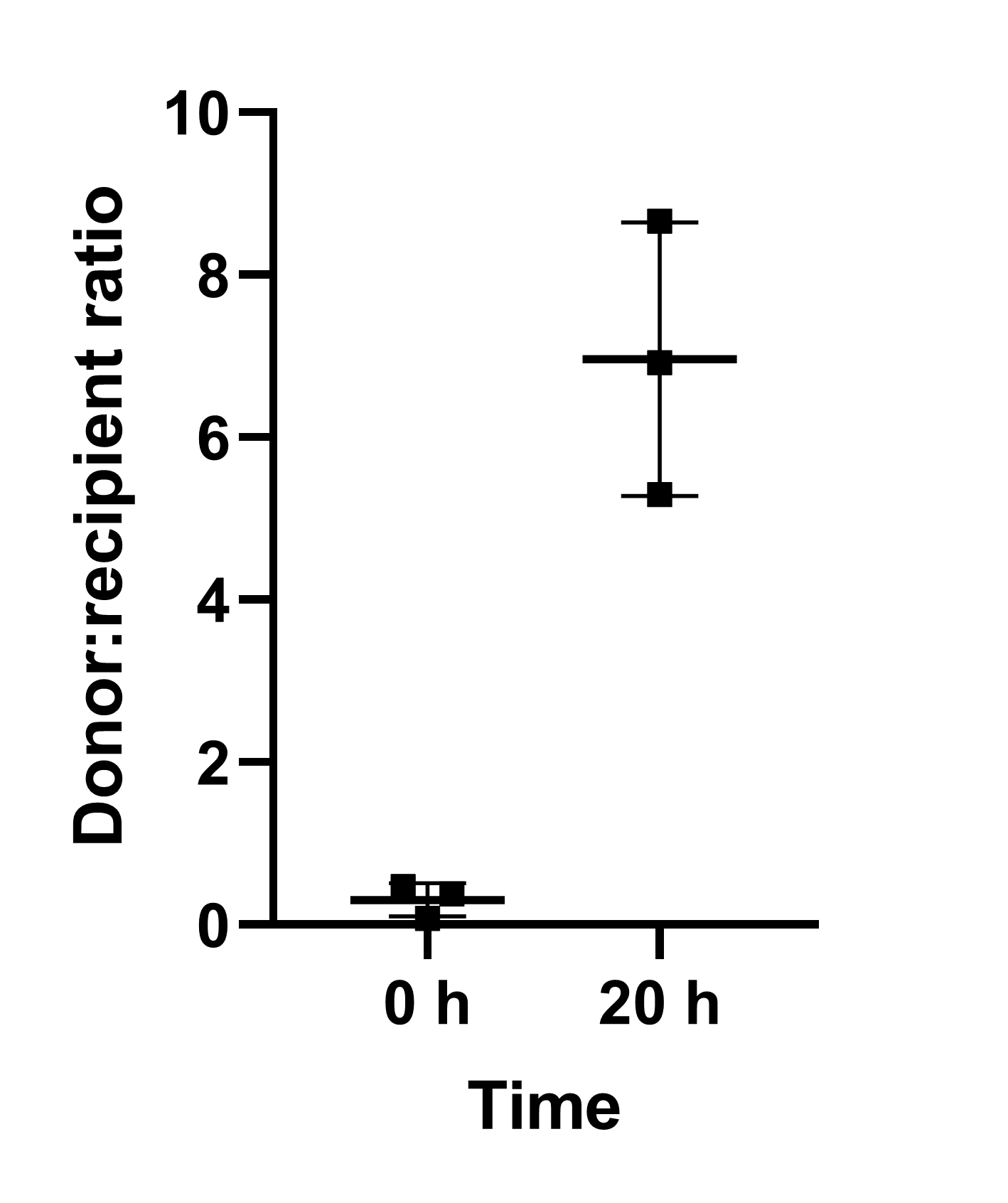

Supplement: Fig. S3 — Mean donor-recipient ratios. [file msphere.00170-23-s0003.tif]

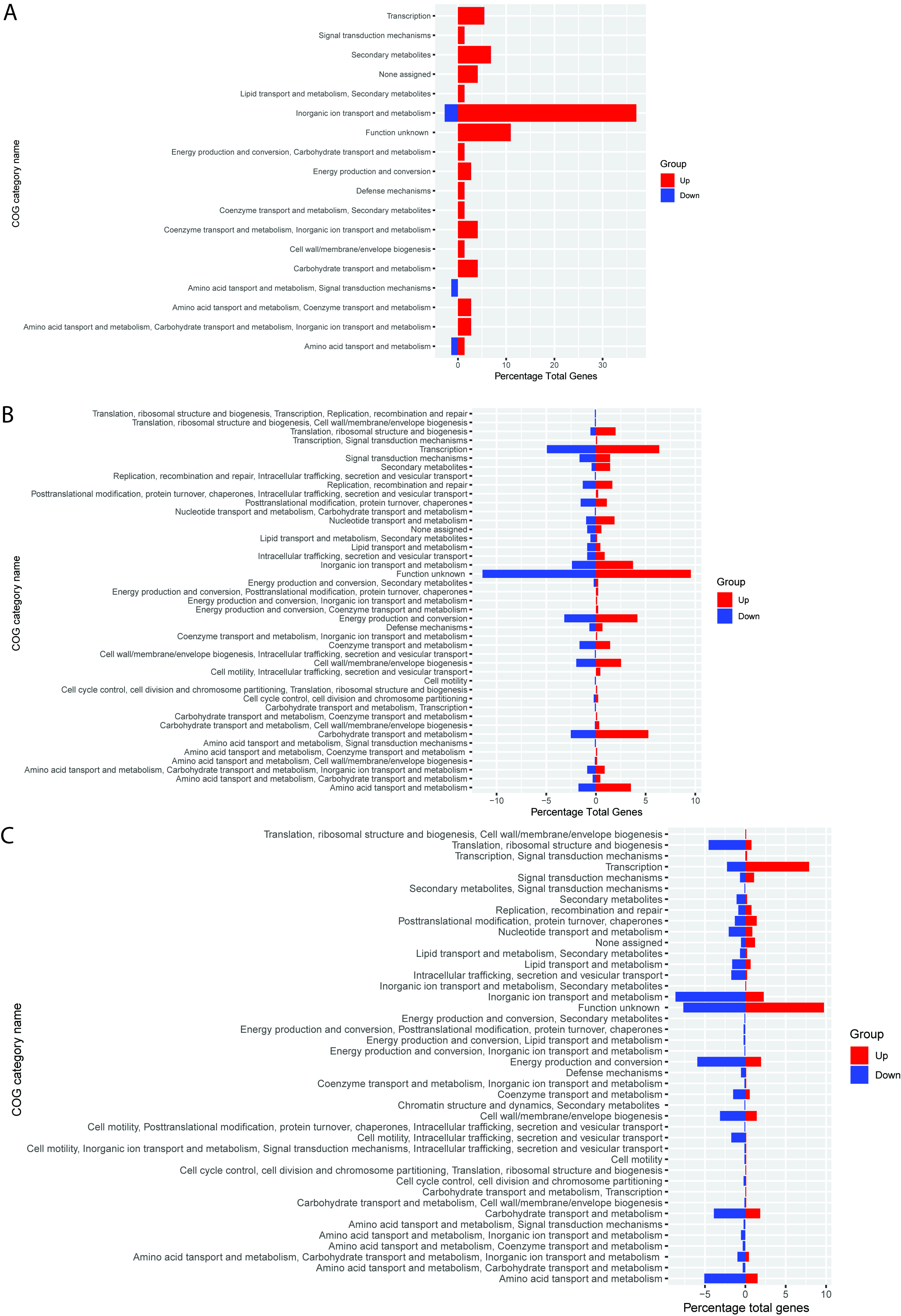

Supplement: Fig. S5 — Plasmid carriage effect on chromosomal gene expression. [file msphere.00170-23-s0005.tif]

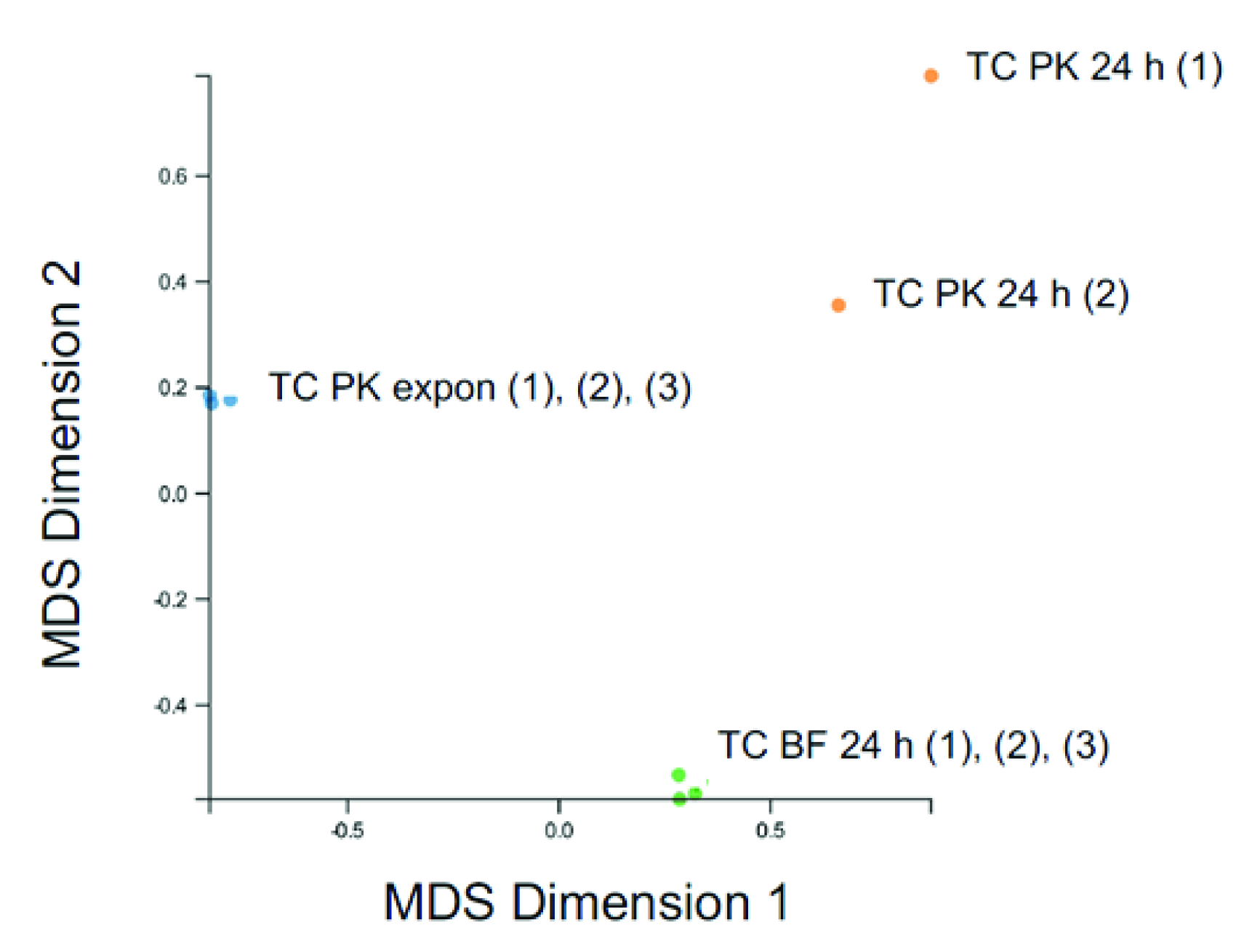

Supplement: Fig. S6 — Multidimensional scaling (MDS) plot. [file msphere.00170-23-s0006.tif]
