# Supplementary material for: Growth in a biofilm promotes conjugation of a blaNDM-1-bearing plasmid between Klebsiella pneumoniae strains
Source: mSphere. 2023 Jul 7;8(4):e00170-23. doi: 10.1128/msphere.00170-23 (PMC10449501; doi:10.1128/msphere.00170-23)
Supplement: Table S1 — Chromosomal genes with altered expression. [file msphere.00170-23-s0007.docx]

**Supplemental Table 1: Chromosomal genes with altered expression across exponential, planktonic 24 h and/or biofilm 24 h conditions**. Statistically significant differential expression of chromosomal genes common to at least two lifestyle conditions, comparing KP20 to the transconjugant (adjusted *P* value = <0.05 and log_2_ fold change mean expression between ≥ 2 and ≤ −2). Blue highlight indicates lower gene expression in the transconjugant compared to KP20, and yellow highlight indicates higher expression in the transconjugant compared to KP20. Empty cells signify the gene/locus tag was absent from the comparison in a given condition.

| **Gene/locus tag** | **Exponential** | **Planktonic 24 h** | | **Biofilm 24 h** | |
| --- | --- | --- | --- | --- | --- |
| KFPKENJG_01701 | -2.27 | -2.61 |  | |  |
| KFPKENJG_01703 | 2.29 | 3.09 | 2.68 | |  |
| *yusV* | 2.74 | -2.59 |  | |  |
| *ttdT* | 2.27 |  | 2.98 | |  |
| *hdfR*_3 |  | -2.18 | 2.01 | |  |
| KFPKENJG_04510 |  | -2.60 | 2.45 | |  |
|  |  |  |  |  |  |
