# Supplementary material for: Growth in a biofilm promotes conjugation of a blaNDM-1-bearing plasmid between Klebsiella pneumoniae strains
Source: mSphere. 2023 Jul 7;8(4):e00170-23. doi: 10.1128/msphere.00170-23 (PMC10449501; doi:10.1128/msphere.00170-23)
Supplement: Table S2 — Primers. [file msphere.00170-23-s0008.docx]

**Supplemental Table 2:** Primers used in this study.

| **Lab ID** | **Description** | **Orientation** | **Sequence (5’-3’)** | **Temp. ◦C** | **Designed by** |
| --- | --- | --- | --- | --- | --- |
| 9 | Generation of hygromycin resistance cassette from pSIM18. Paired with 10. | Forward | ctcgcctttatcggccctcactcaaggatgtattgtggttGATCT GAATTGCTATGTTTA | N/A | S.J. Element |
|  |  |  |  |  |  |
| 10 | Generation of hygromycin resistance cassette from pSIM18. Paired with 9. | Reverse | AGCGGATACATATTTGAATGccgaataacaaagca gagcgcattgtggtgatttatctg | N/A | S.J. Element |
|  |  |  |  |  |  |
| 11 | Check for location of hygromycin resistance cassette insertion in KP1/ check for recipient chromosome in conjugation assays. Paired with 12. | Forward | gatgacaaatgatgaaggaa | 50 | S.J. Element |
|  |  |  |  |  |  |
| 12 | Check for location of hygromycin resistance cassette insertion in KP1/ check for recipient chromosome in conjugation assays. Paired with 11. | Reverse | GGATTTTGGTCATGAGATTA | 50 | S.J. Element |
|  |  |  |  |  |  |
| 15 | Check for *gam* from pACBSCE to confirm presence of pACBSCE recombineering plasmid | Forward | CACTAACCCCCTTTCCTGTT | 52 | S.J. Element |
| 16 | Check for *gam* from pACBSCE to confirm presence of pACBSCE recombineering plasmid | Reverse | GCACCTGTTTGAATCGCTAT | 52 | S.J. Element |
| 17 | Check for *bla*_NDM-1_ from pCPE16_3. Paired with 18. | Forward | GATAGGGGAAGAATTCGAGC | 50 | S.J. Element |
| 18 | Check for *bla*_NDM-1_ from pCPE16_3. Paired with 17. | Reverse | CAATATCACCGTTGGGAT | 50 | S.J. Element |
| 19 | Check for *repA* (FIBK) from pCPE16_3. Paired with 20. | Forward | GACTCATCGGCGGTAAGTTC | 50 | S.J. Element |
| 20 | Check for *repA* (FIBK) from pCPE16_3. Paired with 19. | Reverse | CAGCAGCACCATTGAACTTC | 50 | S.J. Element |
| 21 | Check for *repA* (FIB) from pCPE16_2. Paired with 22. | Forward | GGTCGTATGTTTAGGATAGGA | 50 | S.J. Element |
| 22 | Check for *repA* (FIB) from pCPE16_2. Paired with 21. | Reverse | GCCATACACGGAAATCTGTC | 50 | S.J. Element |
|  |  |  |  |  |  |
| 23 | Check for *repA* (HIB) from pCPE16_2. Paired with 24. | Forward | GGATGGCAGTGACCCTATGG | 50 | S.J. Element |
| 24 | Check for *repA* (HIB) from pCPE16_2. Paired with 23. | Reverse | GGA TCC GTG TCA GTG AGT CG | 50 | S.J. Element |
| 25 | Check for *repA* from pCPE16_4. Paired with 26. | Forward | CGTTCAGTCCGACTGCTGCG | 53 | S.J. Element |
| 26 | Check for *repA* from pCPE16_4. Paired with 25. | Reverse | GGTTCAGTAGAGTTGGCGCT | 53 | S.J. Element |
| 27 | Check for *repA* from pCPE16_5. Paired with 28. | Forward | GAATGGGAGTCTCTTACCGC | 50 | S.J. Element |
| 28 | Check for *repA* from pCPE16_5. Paired with 27. | Reverse | AATACGCAGCAAAGCAATGG | 50 | S.J. Element |
|  |  |  |  |  |  |
| 29 | Check for pCPE16_3 ‘region 1’ (72,750-72,947). Paired with 30. | Forward | GCTTTATTTCCTGCTGTGTC | 50 | S.J. Element |
|  |  |  |  |  |  |
| 30 | Check for pCPE16_3 ‘region 1’ (72,750-72,947). Paired with 29. | Reverse | CCTCAGACATCAGACACTAG | 50 | S.J. Element |
|  |  |  |  |  |  |
| 31 | Check for pCPE16_3 ‘region 2’ (93,043-93,237). Paired with 32. | Forward | CGCTGCATATTTCCTTTATC | 50 | S.J. Element |
| 32 | Check for pCPE16_3 ‘region 2’ (93,043-93,237). Paired with 31. | Reverse | ATTGACCATACAGCCAGGCG | 50 | S.J. Element |
|  |  |  |  |  |  |
| 33 | Check for pCPE16_3 ‘region 3’ (113,401-113,627). Paired with 34. | Forward | CGGGCGGCAGAAGAACAGCA | 50 | S.J. Element |
| 34 | Check for pCPE16_3 ‘region 3’ (113,401-113,627). Paired with 33. | Reverse | GCCACCTGCTGAATCGCCTC | 50 | S.J. Element |
|  |  |  |  |  |  |
| 35 | Check for pCPE16_3 ‘region 4’ (15,027-15,203). Paired with 36. | Forward | ATCACACGCACGGAACTCTA | 50 | S.J. Element |
| 36 | Check for pCPE16_3 ‘region 4’ (15,027-15,203). Paired with 35. | Reverse | CGTGCTAACTTGCGTGATAC | 50 | S.J. Element |
|  |  |  |  |  |  |
| 37 | Check for pCPE16_3 ‘region 5’ (36,129 -36,313). Paired with 38. | Forward | GACGGGGCGGGATTTTTAAG | 50 | S.J. Element |
| 38 | Check for pCPE16_3 ‘region 5’ (36,129 -36,313). Paired with 37. | Reverse | GTCACCCATCCAGCGAAGCA | 50 | S.J. Element |
|  |  |  |  |  |  |
